# The Endocannabinoid System as a Target for Neuroprotection/Neuroregeneration in Perinatal Hypoxic–Ischemic Brain Injury

**DOI:** 10.3390/biomedicines11010028

**Published:** 2022-12-22

**Authors:** Andrea Duranti, Gorane Beldarrain, Antonia Álvarez, Matilde Sbriscia, Silvia Carloni, Walter Balduini, Daniel Alonso-Alconada

**Affiliations:** 1 Department of Biomolecular Sciences, University of Urbino Carlo Bo, 61029 Urbino, Italy; 2 Department of Cell Biology and Histology, School of Medicine and Nursing, University of the Basque Country (UPV/EHU), 48940 Leioa, Spain

**Keywords:** endocannabinoid system, cannabinoid receptors, FAAH inhibitors, MGL inhibitors, neonatal brain injury, hypoxia–ischemia, neuroprotection, neurogenesis

## Abstract

The endocannabinoid (EC) system is a complex cell-signaling system that participates in a vast number of biological processes since the prenatal period, including the development of the nervous system, brain plasticity, and circuit repair. This neuromodulatory system is also involved in the response to endogenous and environmental insults, being of special relevance in the prevention and/or treatment of vascular disorders, such as stroke and neuroprotection after neonatal brain injury. Perinatal hypoxia–ischemia leading to neonatal encephalopathy is a devastating condition with no therapeutic approach apart from moderate hypothermia, which is effective only in some cases. This overview, therefore, gives a current description of the main components of the EC system (including cannabinoid receptors, ligands, and related enzymes), to later analyze the EC system as a target for neonatal neuroprotection with a special focus on its neurogenic potential after hypoxic–ischemic brain injury.

## 1. The Endocannabinoid System

The endocannabinoid (EC) system is a cell-signaling system consisting mainly of at least two cannabinoid (CB) receptors, namely CB_1_ and CB_2_, their endogenous ligands, and the enzymes responsible for the synthesis, transport, and degradation of endocannabinoids (ECs) [1]. Changes in the expression or activity of CB receptors, ligands, or enzymes are implicated in many pathological conditions [2]. Neurological disorders such as anxiety, depression, schizophrenia, neurodegenerative (e.g., Parkinson’s and Huntington’s disease), and stroke-related disorders, together with osteoporosis, multiple sclerosis, neuropathic pain, cancer, glaucoma, hypertension, and obesity/metabolic syndrome are just the major diseases associated with perturbations of the EC system [3]. Recently, it has been hypothesized that CB_2_ receptor activation may be also useful to reduce the inflammatory response induced by SARS-CoV-2 infection, due to its capacity to ameliorate the production of cytokines responsible for the pathological phenomenon [4].

However, changes in EC tone are sometimes transient and likely part of the organism’s compensatory response mainly aimed at reducing symptoms or slowing the progression of pathological conditions. In the nervous system, the activity of the EC system also appears related to neuroprotection, because of its ability to modulate the intensity and extent of a series of dangerous biological events involved in the neurodegenerative process. These include modulation of glutamate excitotoxicity [5] and oxidative stress [6], and a reduction in the inflammatory response [7]. This scenario led to considering the EC system as a potential target for developing new neuroprotective therapies [3]. However, it is not always clear whether an increased activity of the EC system can be consequent to a higher biosynthetic activity or a reduction in the metabolic degradation of the endogenous ligands. Therefore, a better understanding of the role and mechanisms underlying EC tone alterations during the neurodegenerative process represents key factors for developing new therapeutic agents acting through this important modulatory system.

Overall, despite the vast amount of knowledge acquired over time, the exploration of the EC system still represents a stimulating goal. Indeed, the complexity of its structures, the species variability of its characteristics, and the overlapping of pharmacological targets, still leave open many questions and scientific opportunities. This review aims to highlight the potential role of the EC system in the neurodegenerative and neuro-reparative processes resulting from hypoxic–ischemic insults occurring during brain development. A summary of CB receptors, ligands, and related enzymes is also reported.

### 1.1. Cannabinoid Receptors

The effects associated with the endo and exocannabinoid compounds are primarily related to their interaction with the CB_1_ and CB_2_ receptors, discovered some decades ago [8,9,10] and characterized based on their neurobiology signaling [11]. Their involvement in many physiological and pathological events justifies the central role that they play as a possible therapeutic key for many diseases. CB receptors can be stimulated or antagonized by different ligands and can also be modulated through the inhibition of the enzymes responsible for the degradation of their endogenous ligands [12]. Unfortunately, the interaction of exocannabinoids with these receptors, especially with the CB_1_ subtype, is also associated with the psychotropic effects of many recreational drugs, including *Cannabis*, the so-called new psychoactive substances [13], and smart drugs (SPICE and K2…) [14], or to other undesirable serious effects of synthetic agonist or antagonist drugs [15,16,17].

CB_1_ receptors are abundantly expressed in the central nervous system (CNS), particularly in the cerebral cortex, hippocampus, basal ganglia, and cerebellum. CB_2_ receptors, instead, are mostly expressed in the immune system, particularly in B and natural killer cells. However, CB_2_ receptors have been also found in some districts of the CNS [18] and the CB_1_ also peripherally, albeit at low levels [11]. More detailed information on the origin, structural aspects, and signaling processes mediated by CB_1_ and CB_2_ receptors are reported in [19,20].

Generally, the activation of CB receptors determines the inhibition of adenylate cyclase, with a consequent decrease in the levels of cyclic adenosine monophosphate (cAMP), a second messenger involved in numerous intracellular signaling and essential for the regulation of many cell functions. There is also evidence that the CB_1_ receptor, in addition to acting on adenylate cyclase, can be coupled to ion channels [21], confirming the key role of CBs in inducing activation or depression of neurotransmission [11].

Recent studies have also revealed the existence of “atypical” EC receptors, i.e., the transient receptor potential vanilloid (TRPV) channels, involved in the nociceptive signaling; the GRP55, G-protein coupled receptors responsible for some independent CB_1_ and CB_2_ responses; the peroxisome proliferator-activated receptor gamma (PPAR-γ) receptors, which are physiologically involved in glucose metabolism and insulin signaling, and also in inflammation and pain; and the dopamine, adenosine, opioid, and 5-HT_1A_ receptors [22].

### 1.2. Endocannabinoids

Endocannabinoids (ECs) are endogenous lipidic compounds formed by a long-chain polyunsaturated fatty acid tail and a polar head containing functional groups such as amide, ester, ether, or hydroxy one. They bind to CB receptors but, unlike most neurotransmitters that are synthesized and stored in vesicles; their synthesis from membrane phospholipids is on-demand and use-dependent [23,24].

ECs are released from postsynaptic terminals in a Ca^2+^-dependent manner. After their release, they activate presynaptic CB receptors usually through retrograde signaling, although non-retrograde signaling may occur [24]. The retrograde signaling mechanism is responsible for modulating both short-term and long-term neuroplasticity [25]. The short-term type of modulation (seconds) participates in processes, such as depolarization-induced suppression of inhibition and depolarization-induced suppression of excitation. This may occur through the inhibition of Ca^2+^ voltage-gated channels and the modulation of the synaptic release of various neurotransmitters, including glutamate and γ-aminobutyric acid (GABA) [26,27]. In addition, ECs are also involved in long-term synaptic plasticity (in the order of minutes) through a CB_1_ repeated stimulation of these brain circuits [24]. This process leads to the long-term depression phenomenon, with the final decrease in the glutamatergic and GABAergic synaptic activity [28]. Thus, ECs may function as a polymodal signal integrator to allow the diversification of synaptic plasticity in a single neuron [29]. EC receptors, in particular those in the CNS, can, therefore, be potential drug targets for the prevention and treatment of neurologic disorders, such as brain ischemia [30].

**Figure 1:**
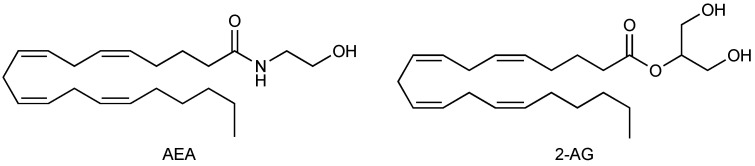
Chemical structures of AEA and 2-AG.

The best-studied ECs are *N*-arachidonoylethanolamine (anandamide and AEA, as seen in Figure 1) [31] and 2-arachidonoyl-*sn*-glycerol (2-AG, as seen in Figure 1) [32,33], but other arachidonic acid derivatives (e.g., noladin ether, virodhamine, and *N*-arachidonoyldopamine) can bind CB_1_ and/or CB_2_ receptors, although their physiological role is not yet clear.

AEA is a full or partial agonist of the CB_1_ receptor but also shows low activity towards the CB_2_ receptor [34,35,36,37,38], whereas 2-AG is a full agonist of both CB_1_ and CB_2_ receptors [39].

Differences between AEA and 2-AG occur also in the biosynthetic pathways responsible for their formation and degradation. With reference to the synthetic step, *N*-acyl phosphatidylethanolamine phospholipase D (NAPE-PLD) [40,41] or diacylglycerol lipase (DGL) [42,43] are the enzymes directly involved, whereas fatty acid amide hydrolase (FAAH) [44,45,46,47] or monoglyceride lipase (MGL) [48,49,50] are the main enzymes responsible for their metabolism, leading to the formation of arachidonic acid and ethanolamine [51] or glycerol [52] following a cellular internalization process carried out by specific transporters [53,54,55].

### 1.3. Cannabis, Phytocannabinoids, and Synthetic Cannabinoids

*Cannabis* contains more than 500 compounds, of which at least 100 are known to be phytocannabinoids [56] owing to pharmacological properties [57]; they are described in detail in [58]. Paleobotanical studies attest that *Cannabis* was already present during the Holocene epoch about 11,700 years ago, more likely in the territories of Central Asia near the Altai Mountains [59]. The first written testimony on the use of *Cannabis* for therapeutic purposes dates back to 2700 BC, when the Chinese emperor Shen-Nung reported a detailed description of it in a book that later became the Chinese compendium of drugs [60]. Chinese people used this plant for diseases such as rheumatic pain, malaria, constipation, etc. [61]. Despite their long history, the phytocannabinoids contained in *Cannabis* were identified only a few decades ago and progressively studied both as single molecules and as their derivatives, and also based on structure–activity relationship studies [62].

**Figure 2:**
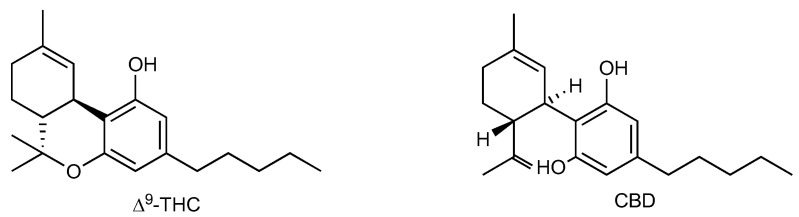
Chemical structures of Δ^9^-THC and CBD.

The most studied and characterized phytocannabinoids have been Δ^9^-tetrahydrocannabinol (Δ^9^-THC, Figure 2) [63], which constitutes the main psychoactive compound of *Cannabis*, and the non-psychotropic cannabidiol (CBD, Figure 2) [64]. Despite similar structures, their pharmacodynamic properties deeply differ.

Δ^9^-THC acts as a partial agonist of the CB_1_ receptor, which explains its strong psychoactive outcomes inducing the tetrad effects (hypothermia, catalepsy, hypolocomotion, and analgesia). These unwanted effects make the medical use of the Δ^9^-THC strongly restricted despite its beneficial action (neuroprotective, anti-inflammatory, and antispasmodic), which depends on the activation of CB_2_ and PPAR-γ receptors [56]. In addition, the activity of the molecule is dependent on cell type, receptor expression, and the presence of ECs or other agonists [56,65].

For its part, CBD has a high affinity on a series of targets, including CB_1_ and CB_2_, GPR55, TRPV, PPAR-γ, 5-HT_1A_, dopamine, and opioid receptors, and also on ion channels, which contributes to the beneficial effects of *Cannabis* in diseases related to a wide range of pathologies (neurological, ischemic stroke, inflammatory, pain, etc.) [22,66,67].

Although Δ^9^-THC and CBD are the best-known and studied phytocannabinoids, other compounds have been relieved in *Cannabis* owing to their therapeutic potential. Among these, the main ones are reported below. (1) Cannabigerol (CBG), which is a non-psychotropic derivative with a low affinity for the CB_1_ and CB_2_, but it is able to interact with other receptors, such as α_2_-adrenergic, TRPs superfamily, and 5-HT_1A_, and to possess various properties (antiproliferative, antibacterial, antioxidant, etc.) [56]. (2) Cannabichromene (CBC), which acts weakly with CB_1_ and CB_2_ receptors and is able to inhibit AEA uptake, is the most potent agonist of TRPA1 channels and possesses antinociceptive and anti-inflammatory properties in vitro and in vivo [56]. (3) Δ^9^-Tetrahydrocannabivarine (Δ^9^-THCV), which is the *n*-propyl analogue of Δ^9^-THC and acts weakly with CB_1_ receptors and more potently with the CB_2_ ones. However, it is also able to act with other targets such as TRP, GPR6, GPR55, and D_2_ receptors and to exert related pharmacological actions [56]. (4) Cannabinol (CBN), which is the first phytocannabinoid structurally characterized; its derivatives are considered as the oxidative by-product of the degradation process of Δ^9^-THC and CBD [62,68]. It acts as a partial agonist of CB_1_ and CB_2_ and exerts neuroprotective, antiepileptic, and analgesic properties [68,69].

Synthetic cannabinoids are ligands that bind to CB receptors and modulate their activity. Their design and the studies aimed to acquire information on the structural requirements to establish interactions with CB receptors. Moreover, the goal was also related to the understanding of the role played by molecules that bind with these targets, the role of the targets themselves, and, more generally, the role of the EC system, since synthetic cannabinoids can be considered tools to increase knowledge in the field. As for natural ligands, the role of synthetic cannabinoids must always be contextualized within the situation of a risk/benefit ratio that would derive from their use. In spite of the large effort in synthesizing and characterizing the pharmacological profile of these molecules, apart from nabilone, there are currently no drugs on the market containing a synthetic cannabinoid, but many of them are used for recreational purposes and are included in the list of substances of abuse [15,70,71,72,73].

The structural requirements that allow synthetic cannabinoids to interact with CB receptors are highly variable, a situation that strongly influences their pharmacological activity, in particular for what concern agonism, antagonism, and, more rarely, inverse agonism. It is interesting to consider, however, that their activity sometimes depends on the experimental model used for their characterization (e.g., antagonism vs. inverse agonism). An overview of these features and the therapeutic potential of CB ligands are presented in Refs. [74,75,76,77,78,79].

The therapeutic interest of the drugs that bind to CB receptors is also proved by the marketed drugs mentioned above. Cesamet^®^ (nabilone—the synthetic dibenzopyran-9-one analog of Δ^9^-THC), administered for the improvement of chemotherapy-induced nausea and vomiting (CINV) states in patients not responding to conventional antiemetic therapies. Marinol^®^ (dronabinol—the synthetic pure isomer (–)-*trans*-Δ^9^-THC) is prescribed for the same purposes as the former and also for appetite stimulation in patients with AIDS (acquired immune deficiency syndrome). Sativex^®^ (Δ^9^-THC and cannabidiol in an approximate 1:1 fixed ratio) is used for the symptomatic relief of the pain and/or the management of neuropathic pain and spasticity in adults with multiple sclerosis and is not responsive to other antispasticity therapies. More recently, a new drug containing > 98% CBD and less than 0.15% Δ^9^-THC (Epidiolex^®^) has been approved for the treatment of seizures associated with two rare and severe forms of epilepsy (Lennox–Gastaut and Dravet syndromes) in patients two years of age and older [56,66,80,81].

**Figure 3:**
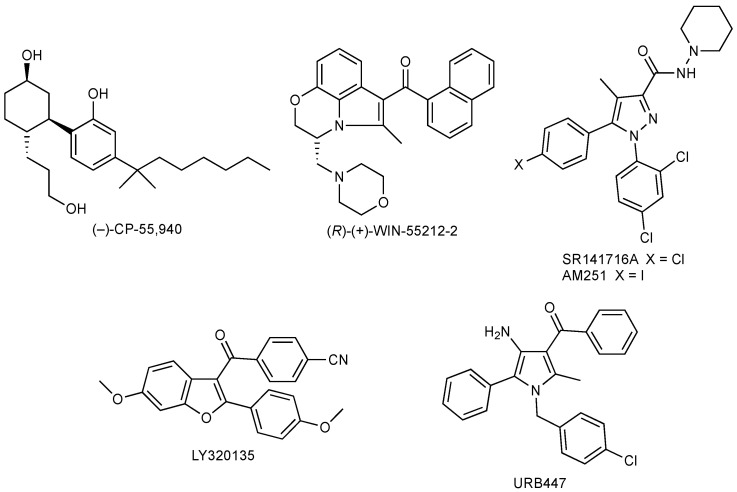
Chemical structures of neuroprotective CB ligands.

An intriguing and interesting feature of CB receptor ligands is that they are able to exert a neuroprotective role after ischemic injuries [82,83,84,85]. The CB_1_ and CB_2_ agonists (–)-CP-55,940 [86] and (*R*)-(+)-WIN-55212-2 [87], the CB_1_ inverse agonists SR141716A [88] and AM 251 [89], the CB_1_ antagonists LY320135 [90], and the CB_2_ agonist/CB_1_ antagonist URB447 [91] (Figure 3) are the related tools studied.

Despite the therapeutic use of drugs containing *Cannabis* and its derivatives or of synthetic ligands of CB receptors, problems related to their abuse remain open. For this reason, molecules inhibiting the degradation of endogenous ligands may represent an interesting alternative for modulating the EC system [92].

### 1.4. FAAH and MGL Inhibitors

These compounds increase AEA and 2-AG levels by inhibiting their intracellular degradation. The hypothesis leading to the design and development of EC metabolism inhibitors is based on the fact that, by blocking the degradation of these endogenous mediators, we can increase their concentrations in the physiological districts where they are formed. By using this approach, it may be possible to obtain pharmacological agents characterized by the absence of the psychotropic side effects typical of CB_1_ exogenous ligands. Indeed, ECs are synthesized and released *on-demand* in a tissue-specific and time-dependent manner and inhibitors of their metabolic enzymes will cause an increase in EC levels only where and when it is physiologically required. In this way, the activation of CB receptors is obtained through endogenous ligands, but it is prolonged over time. Several pieces of evidence support this approach. For example, it has been reported that FAAH knockdown mice show increased levels of AEA in the brain and other tissues leading to CB_1_ receptor-mediated analgesia [93,94], or a reduction in anxiety symptoms without the appearance of catalepsy [95].

**Figure 4:**
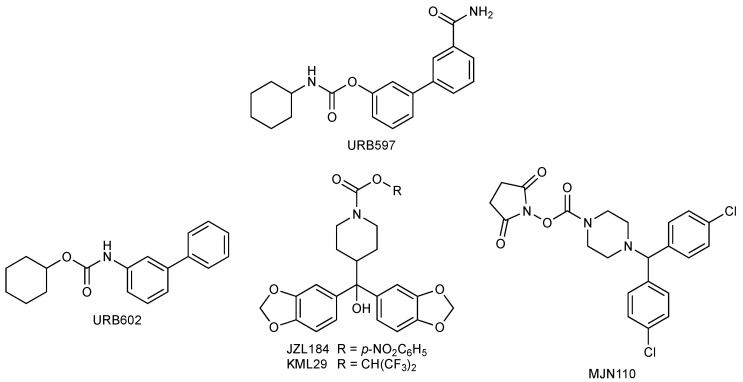
Chemical structures of neuroprotective FAAH and MGL inhibitors.

The possibility of targeting the FAAH and MGL enzymes, therefore, may represent an important therapeutic approach for different pathologies [2,96,97,98,99] and, even if there are no drugs on the market yet, studies carried out in this regard are promising. Disorders related to anxiety, pain, and cigarette and cannabis smoking are the main pathological states in which EC metabolism inhibitors have been studied [2], and clinical studies are in progress. FAAH and MGL inhibitors have also been considered pharmacological tools to increase information on the role of the EC system in neurodegeneration/neuroprotection after ischemic injuries [100,101,102,103,104,105]. The FAAH inhibitor URB597 [95,106,107,108,109,110,111,112,113] and MGL inhibitors URB602 [101,114,115,116], JZL184 [117], KML29 [118], and MJN110 [119] (Figure 4) are the main experimental molecules assessed in these studies.

## 2. The Endocannabinoid System in Prenatal and Postnatal Development

During prenatal and postnatal brain development, the EC system may play an active role in the control of the cell cycle, proliferation, survival, and differentiation of neural stem cells [120], as well as in the maturation of the nervous system and its functions. The modulation of some of these processes appears regulated by the CB_1_ receptor, which is expressed in the very early stages of neural development. Indeed, the expression of members of the EC system has been described during early developmental and postnatal stages [121,122,123], and in the embryonic rat brain, its presence was found around day 11 of gestation [124]. In 1998, Berrendero et al. [121] not only demonstrated the existence of CB_1_ receptors, but they also showed that these receptors were already functional in embryonic stages. In humans, the presence of CB_1_ receptors has been documented as soon as at week 14 of gestation in the embryo [125].

The regions in which CB_1_ receptors are expressed in these early stages, i.e., the corpus callosum, stria terminalis, stria medullaris, fasciculus retroflexum, or anterior commissure, are related to processes such as cell proliferation, migration, axonal elongation and synaptogenesis [121,122,123,126]. The later modifications in CB_1_ receptors’ location during neural development (becoming different in the adult brain), suggest that their expression in the brain changes once their contribution to neural development finishes [121,123].

In murine cell cultures, CB_1_ receptors appear in several cell types, including stem-like cells, astrocytes, and immature neurons [127]. It has also been observed that the agonist (*R*)-(+)-methanandamide promoted self-renewal, multipotency, and neuronal differentiation via CB_1_ activation. When ECs are produced or exogenously administered with bind CB_1_ receptors, the α_i_ subunit linked to the protein inhibits the activity of adenylyl cyclase and the synthesis of cAMP. Low levels of cAMP reduce the activity of the protein kinase-A and, consequently, the type-A potassium channels are activated and lead to membrane hyperpolarization. The α_0_ subunit of the G protein associated with the CB_1_ receptor, instead, inhibits voltage-dependent Ca^2+^ channels causing cell depolarization. The β and γ subunits, moreover, interact with pathways, such as PI3K or PKB/Akt, that have been shown to induce the expression of transcription factors associated with cell proliferation (CREB, STAT-3, PAX-6, and β-catenin). CB receptors are also closely related to neutral sphingomyelinase, which generates ceramide from sphingomyelin located in the plasma membrane, thus activating the synthesis of transcription factors, such as ERK or p38, that control cell fate and survival [128,129]. The involvement of factors, such as ERK or PI3K, in neurogenesis associated with CB_1_ activation was also observed by Xapelli et al. [127].

The processes of migration and path-finding during neurogenesis appear also partially regulated by the CB_1_ receptor. Their blockage with a selective antagonist caused a decrease of 50% in migration in a scratch wound assay in mouse fetal cortex-derived cells [130]. The same authors also labeled the rostral migratory stream explants embedded in Matrigel using the migrating neuroblast markers PSA-NCAM and DCX, and observed a significant reduction (30%) in the migratory distance after the treatment with a CB_1_ receptor antagonist. The role of the EC system in the path-finding function also became evident when EC signals were proven to be behind axon direction cues, helping neurons find their path [131].

Together with CB receptors, the ECs AEA and 2-AG also make their appearance in the prenatal period. Despite the presence of AEA levels having been detected from the early stages of the embryo [132], 2-AG seems to be predominant in the fetal period, as this molecule has been found in higher concentrations than AEA [133]. Conversely, AEA levels increase gradually during brain development until an adult level is reached, while the concentration of 2-AG remains more or less stable than in the fetal, young, and adult brains [122].

## 3. The Endocannabinoid System as a Target for Neuroprotection in Hypoxic–Ischemic Encephalopathy

Perinatal HI leading to neonatal encephalopathy (NE) represents a major cause of death and long-term disability in neonates [134]. Each year, up to 20,000 infants are affected by NE in Europe and even more in regions with a lower level of perinatal care [135]. Whereas the incidence of NE in Western Europe and North America is around 1.6/1000 term births [136], neonatal mortality is 6 times higher in developing or low-resourced countries compared with developed or middle-to-high-resourced countries.

Current treatment options for HI are extremely limited, making the management of long-term outcomes or its prevention difficult. Actually, the only approved therapy is therapeutic hypothermia, consisting in lowering the body temperature of patients to 33.5 °C for 72 h through cooling of either the whole body or just the head [137]. Therapeutic hypothermia is routinely implemented in the majority of first-world hospitals to treat term infants with moderate to severe NE; however, cooling is only partially effective as a neuroprotective therapy (>45% of infants have adverse neurodevelopmental outcomes despite treatment) [138]. At the same time, hypothermia can develop some potential side effects due to the slowing of the mechanisms of clearance and metabolism, the induced immunosuppressive activity, and the increase in energy expenditure resulting from the thermoregulatory response [139]. As the current cooling therapy protocols appear to be optimal [140], there is an urgent need to improve neonatal neuroprotection by developing additional safe and effective neuroprotective treatments [141,142].

The EC system is able to limit the deleterious effects caused by multiple toxic stimuli such as glutamate excitotoxicity, oxidative stress, and inflammation, thus providing neuroprotection in different paradigms of brain injury [84,143]. Therefore, compounds that modulate the EC system could be promising neuroprotective and/or neurogenic agents for the treatment of CNS pathologies, including NE.

The first cannabinoid tested in cerebral ischemic models was the synthetic CB_1_/CB_2_ agonist (*R*)-(+)-WIN-55,212-2 [144]. The authors showed that the exogenous administration of this CB agonist significantly reduced the infarct volume and the loss of hippocampal neurons. They also studied the neuroprotective effect of cannabinoids during brain development and showed that exogenous administration of the ECs AEA and 2-AG reduced brain infarction in newborn rats subjected to HI [145]. Later, some of the co-authors described the neuroprotective and long-lasting beneficial effect of URB602, an inhibitor of the degradation of 2-AG [101] in the same murine model. The neuroprotective effect of cannabinoids was also confirmed in an experimental model closer to the human condition, i.e., in the fetal lamb. In this model, the synthetic cannabinoid agonist (*R*)-(+)-WIN-55,212-2 protected the neonatal brain at very low doses to maintain mitochondrial integrity and functionality [146], to reduce apoptotic cell death [147], and to ameliorate the inflammatory response [148].

The classical way to modulate the EC system is through the activation or blockade of CB_1_ and CB_2_ receptors, as described in the first part of this review. However, CB_1_ receptors seem to play a dual role in post-ischemic neuronal damage, as the decrease in glutamate release due to CB_1_ activation is accompanied by a parallel decrease in GABA release, resulting in neurotoxicity instead of neuroprotection [149]. Moreover, CB_1_ overactivation in the perinatal period could be harmful [150] and this can limit the translational interest of CB_1_ agonists. In addition, CB_1_-mediated psychoactive effects [151], which are unwanted in clinical treatments, should also be considered.

Activation of the other CB receptor, the CB_2_, results in potent anti-inflammatory effects [143], and the CB_2_ antagonism has no described beneficial effect. A therapeutic approach with drugs interacting with CB_2_ receptors can be developed using either indirect (e.g., cannabidiol) or selective (e.g., GW405833) CB_2_ agonists. Nevertheless, cannabidiol can induce severe hypotension [152] despite being neuroprotective in different experimental paradigms [153], whereas GW405833 showed no protection after HI [154]. This evidence together with the finding that the CB_1_ antagonist/inverse agonist rimonabant also exerts a neuroprotective effect, which adds further complexity to the effect of cannabinoid-interacting compounds in neurodegeneration.

Recently, some of the co-authors evaluated the neuroprotective potential of the synthetic cannabinoid URB447 [85]. URB447 is the first mixed CB_1_ antagonist and CB_2_ agonist that binds to both CB_1_ and CB_2_ receptors with submicromolar affinity and good stereoselectivity [91]. URB447 strongly reduced brain injury when administered before HI in neonatal rats, but more interestingly, the compound was effective also when administered 30 min or 3 h after the initial insult. URB447 reduced cerebral infarction by 95.7% (30 min) and 88% (at 3 h) in the whole ipsilateral (damaged) hemisphere.

Since a pharmacological intervention within 3 h after the injury is considered a clinically feasible therapeutic window to treat perinatal brain injury in humans [155], we characterized the effect of URB447 administered at this time point, focusing on the consequences of HI and URB447 administration on the activation of glial cells and white matter injury. Together with a reduction in astrogliosis and microglial activation, URB447 decreased white matter damage restoring myelin basic protein levels 7 days after HI, confirming the important role played by the EC system in the neurodegenerative and neuroreparative processes after HI.

As commented above, nowadays, the only clinical therapy against HI-induced NE is moderate hypothermia, which exerts a number of neuroprotective responses through the reduction in excitotoxicity, free radical exposure, blood–brain barrier dysfunction, and delayed cell death [156]. Leker et al. [157] observed that a single injection of the CB_1_ synthetic agonist HU-210 significantly reduced body temperature, conferring a strong neuroprotective effect to the hypoxic–ischemic rats, a beneficial effect that was lost when animals were treated with the selective CB_1_ antagonist SR141716. The enhancement of hypothermia by stimulating the EC system or by the combined therapy EC system plus hypothermia may have beneficial outcomes in neonates, so these responses are currently under investigation in preclinical models [158,159].

## 4. Can Endocannabinoid System Interacting Drugs Modulate Neurogenesis after HI?

The discovery of stem cells in the postnatal and adult mammalian brain changed the previously believed assertion that the adult brain is unable to replace lost neurons [160,161]. Although still unknown with certainty in other regions of the CNS, two neurogenic areas persist after birth: the subventricular zone (SVZ) of the lateral ventricles and the subgranular zone (SGZ) of the dentate gyrus of the hippocampus [162,163,164].

The ability to generate new neurons and glial cells from these niches may contribute to the plasticity of the newborn brain and tissue remodeling after damage [165,166,167,168,169]. Based on their regenerative potential, cells from the SVZ of the ventricles can be molecularly manipulated in situ to induce their proliferation and migration to damaged sites or stimulated in vitro for later transplantation [162,170,171,172,173]. However, the processes of proliferation, migration, differentiation, and survival will depend on a wide range of factors, including the type, intensity, duration, and/or location of the damage [174]. Thus, it is not yet known whether these newly formed neurons are properly integrated into the existing neural network and if they can represent a fully functional microenvironment after a brain injury [175]. It has been estimated that 85% of the new neurons generated in response to the insult do not survive after reaching maturation [176].

The global damage induced by perinatal HI may also affect the neurogenic niches and their neuro-proliferative capacity. After 24–48 h from moderate/severe hypoxic–ischemic damage, the SVZ may show extensive cell death, primarily affecting neuronal stem cells and also oligodendrocyte progenitors [177,178]. Interestingly, the neurogenic potential of this area can be affected independently from cell death [179]. Indeed, in a preclinical model close to the human condition, i.e., newborn piglets, it has been shown that a decreased cellularity is associated with a reduction in cell proliferation and neurogenesis in the SVZ [179]. These effects occurred without necrotic or apoptotic cell death 48 h after hypoxic–ischemic damage. Whether this discrepancy could be related to differences in the severity/duration of the insult or the experimental model employed (rodent vs. piglet) remains the subject of investigation. It should also be considered that the SVZ can present sub-regional sensitivity, with areas and cell types showing selective vulnerability to the insult. Higher rates of survival were observed in its medial zone [180] and in different responses of pre-oligodendrocytes and neuroblasts to hypoxic–ischemic damage [181].

Whereas several works pointed toward HI leading to decreased cell proliferation in the SVZ (for a review, see [182]), other authors have described that the injured ipsilateral SVZ has the ability to increase its size after a longer recovery interval [176,178,183,184], a phenomenon attributed to increased cell proliferation [176,185]. For its part, the undamaged contralateral SVZ can also suffer an expansion after HI [176], with the most undifferentiated precursors being responsible for this increase in its size [185].

The other neurogenic niche, the SGZ of the dentate gyrus of the hippocampus, also revealed conflicting results. Bartley et al. [186] showed that neuronal (together with microglial and endothelial) cell proliferation was significantly increased in the injured ipsilateral hippocampus. The authors used a postnatal day 7 (P7) neonatal mice model subjected to permanent unilateral carotid ligation plus 8% hypoxia for 75 min. Conversely, early after the publication of that work, Kadam et al. [187] described that total counts of new cells were significantly lower in both ipsilateral and contralateral hippocampi, which in turn correlated with lesion-induced atrophy. They used, however, a neonatal stroke model of unilateral carotid ligation alone to produce infarcts in P12 CD1 mice. In a more recent work by Ziemka-Nalecz et al. [188] using the HI permanent unilateral carotid ligation plus hypoxia (7.6% O_2_ for 60 min) model of brain injury, the authors showed no signs of increased or decreased cell proliferation in the dentate gyrus of the hippocampi, with no differences between sham (non-operated), ipsilateral, or contralateral hippocampi, in none of the five timepoints evaluated (3, 6, 9, 11, or 14 days after HI). This usage of different experimental models of brain injury may add complexity to unraveling the neurogenic response of the neonatal hippocampal SGZ.

To better understand the modulation in cellular populations after HI, experiments have been carried out using flow cytometry with multi-markers in order to quantify the proportion of each cell type. It seems that neuronal stem cells decrease while multipotential, as well as the glial cell progenitors, increase [189]. The increase in the number of reactive astrocytes can be translated into greater production of components of the extracellular matrix, such as hyaluronic acid and chondroitin sulfate, which can, in turn, inhibit the differentiation of oligodendrocytes and limit myelin synthesis [190,191]. This suggests that hypoxia–ischemia may alter the cellular composition of the neurogenic niches [189].

As described, the effect of neonatal HI on the neurogenic response after brain injury remains far from clear [179]. The modulation of the proliferative capacity of the neurogenic niches might be enhanced by using CBs, as the EC system seems to play an important role in processes, such as cell proliferation and differentiation of neural stem cells, during normal brain development.

Aguado et al. [192] observed that stimulation of the EC system enhanced neurogenesis after kainic acid-induced excitotoxicity in neural progenitor cell cultures. The effect was revealed as increased expression of the progenitor markers nestin, Sox-2, and musashi-1, and also as a higher proliferation rate. They also examined whether neural progenitor cell division may result in effective neurogenesis. By immunostaining dividing cells with the mature neuron marker NeuN, the authors described the presence of newly generated neurons one month after injury, suggesting that long-term neurogenesis can be enhanced by EC system modulation [192].

In a model of HI in rodents, Fernández-López et al. [193] stimulated the CB_1_ receptor by exogenous administration of the synthetic cannabinoid (*R*)-(+)-WIN-55,212-2, showing increased cell proliferation and doublecortin expression (a marker of neuroblasts) after HI and cannabinoid administration. However, the long-lasting effect described by Aguado et al. [192] here was lost. (*R*)-(+)-WIN-55,212-2 was able to promote neurogenesis up to 7 days after HI (P14), but the survival of the new neurons decreased shortly after the withdrawal of the treatment. It remains unclear whether prolonging the administration of cannabinoids could be beneficial regarding neurogenesis [193].

Therapeutic hypothermia (the only clinical therapy against HI-induced neonatal encephalopathy) is also able to modulate and enhance endogenous reparative processes. Bregy et al. [194] showed an increase in doublecortin-positive cells in the hippocampal dentate gyrus of cooled animals treated with therapeutic hypothermia after experimental traumatic brain injury. Works using models of ischemic and hypoxic–ischemic brain injury described similar results [195,196]. Rats treated with hypothermia increased their counts of neurogenesis markers compared to normothermic animals. As the activation of the EC system may decrease body temperature, it seems feasible that exogenous administration of CBs may indirectly modulate the neurogenic response after neonatal brain damage.

In addition to neurogenesis, cannabinoids may be of great benefit in white matter recovery after brain damage. The administration of the CB_1_ agonist ACEA resulted in increased Olig2 (an oligodendrocyte progenitor marker) expressing cells in the SVZ andmyelination in the subcortical white matter [197]. (*R*)-(+)-WIN-55,212-2 administration after HI also promoted remyelination of the injured external capsule by increasing the number of early oligodendrocyte progenitors and mature oligodendrocytes [193]. The enhancement of oligodendrogenesis is of great interest when treating the developing brain, as increased remyelination is linked with the improvement of sensorimotor functions after hypoxic–ischemic injury [198].

## 5. Concluding Remarks and Perspectives

The ubiquitous lipid signaling-based EC system is involved in outstanding regulatory functions throughout the human body, including neural development under physiological conditions and neuroprotection, and repair after pathophysiological processes.

In the context of neonatal brain injury, the administration of endogenous or exogenous CBs, or the blockage of EC degradation, has revealed a strong neuroprotective response in different preclinical models after HI. Similarly, the possibility of tissue repair in the developing brain by enhancing the proliferative potential of the SVZ and SGZ neurogenic niches is currently under active investigation. Selective modulation of the EC system in the sites of damage by targeting the enzymes responsible for EC degradation may represent an important therapeutic approach in order to avoid non-desired widespread effects.

Despite the clinical use of CB-related drugs that must be taken with caution, the modulation of the EC system to ameliorate the neurological consequences after neonatal HI is currently an exciting field of research with enormous possibilities for clinical translation.

## Data Availability

Data sharing not applicable.
